# Supplementary material for: The Construction and Exploration of a Comprehensive MicroRNA Centered Regulatory Network in Foxtail Millet (Setaria italica L.)
Source: Front Plant Sci. 2022 May 6;13:848474. doi: 10.3389/fpls.2022.848474 (PMC9121102; doi:10.3389/fpls.2022.848474)
Supplement: Supplementary file 1 [file Data_Sheet_1.zip › Sit-miR3979_Seita.7G087900.1_213_TPlot.pdf]

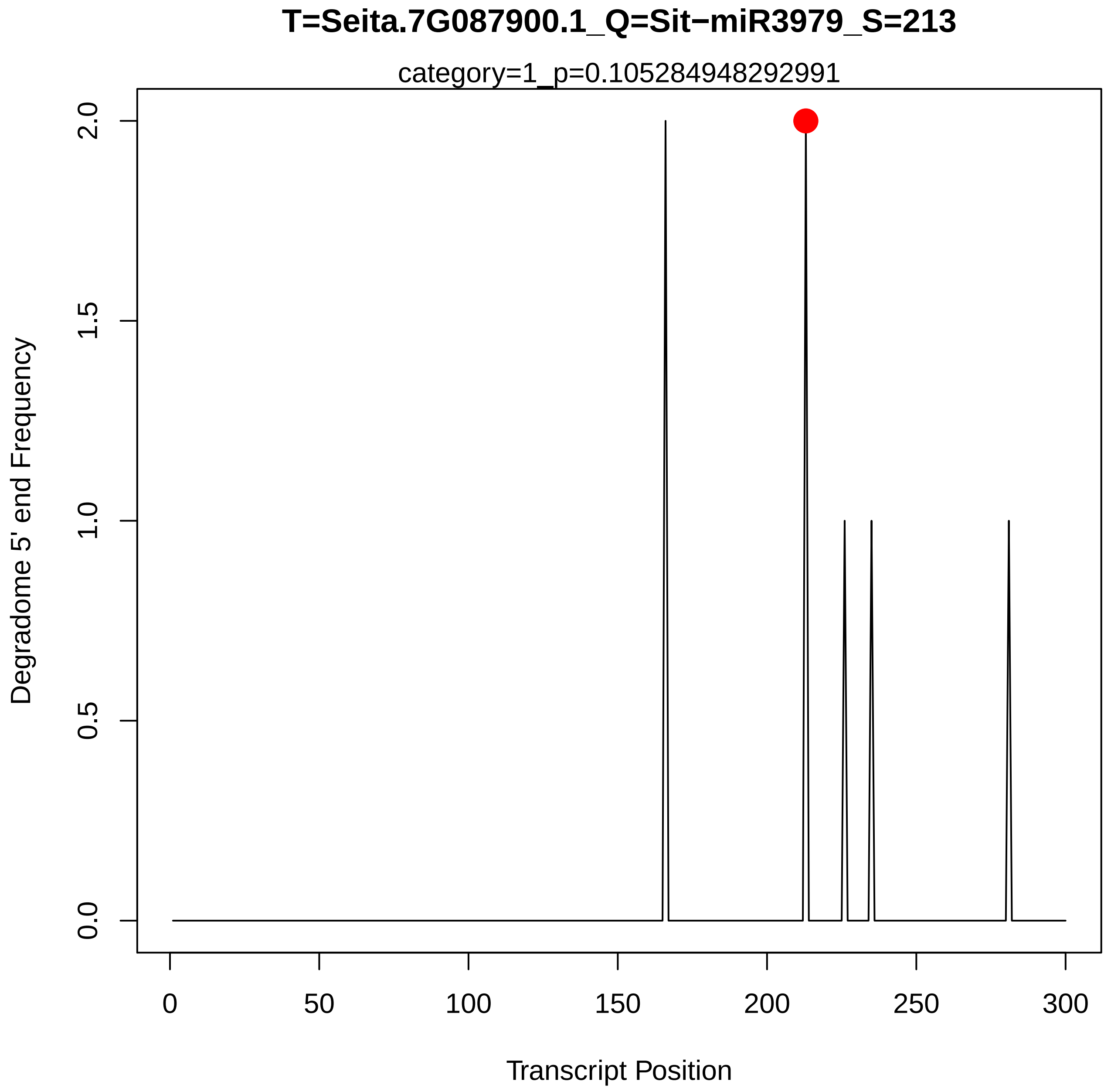
T=Seita.7G087900.1_Q=Sit−miR3979_S=213
category=1_p=0.105284948292991
Degradome 5' end Frequency
0.0
0.5
1.0
1.5
2.0
0
50
100
150
200
250
300
Transcript Position
